# 3D geometry of femoral reaming for bone graft harvesting

**DOI:** 10.1038/s41598-021-95983-8

**Published:** 2021-08-25

**Authors:** Dominic Gehweiler, Nina Schmitz, Boyko Gueorguiev, Ivan Zderic, Leonard Grünwald, Geoff Richards, Dirk Wähnert, Michael J. Raschke

**Affiliations:** 1 AO Research Institute Davos, Clavadelerstrasse 8, 7270 Davos, Switzerland; 2 Department of Trauma, Hand and Reconstructive Surgery, University Hospital Münster, Albert-Schweitzer-Campus 1, Building W1, 48149 Münster, Germany; 3 Department of Trauma Surgery and Orthopaedics, Protestant Hospital of Bethel Foundation, University Hospital OWL of Bielefeld University, Campus Bielefeld-Bethel, Burgsteig 13, 33617 Bielefeld, Germany; 4 Department of Traumatology and Reconstructive Surgery, BG Traumacenter Tübingen, University of Tübingen, Schnarrenbergstraße 95, 72076 Tübingen, Germany

**Keywords:** Preclinical research, Translational research

## Abstract

The reamer-irrigator-aspirator (RIA) technique allows to collect large bone graft amounts without the drawbacks of iliac crest harvesting. Nevertheless, clinical cases with occurrence of femur fractures have been reported. Therefore, this study aimed to systematically investigate the three-dimensional geometry of the reamed bone as a function of the reaming diameter and its influence on the associated potential fracture pattern. Forty-five intact fresh-frozen human cadaveric femora underwent computed tomography (CT). They were randomized to three groups (n = 15) for reaming at a diameter of either 1.5 mm (Group 1), 2.5 mm (Group 2) or 4.0 mm (Group 3) larger than their isthmus using RIA. Reaming was followed by a second CT scan, biomechanical testing until failure and a third CT scan. All CT scans of each femur were aligned via rigid registration, and fracture lines were visualized. Subsequently, a decrease in wall thickness, cross-sectional area, and harvested bone volume have been evaluated. The total volume of the bone graft was significantly higher for Group 3 (7.8 ± 2.9 ml) compared to Group 1 (2.9 ± 1.1 ml) and Group 2 (3.0 ± 1.1 ml). The maximal relative decrease of the wall thickness was located medially (72.7%) in the third (61.4%), fourth (18.2%) and second (9.1%) eighth for all groups. As the diameter of the reaming increased, an overlap of the fracture line with the maximal relative decrease in wall thickness and a maximal average relative decrease of the cross-sectional area became more frequent. This suggests that a reaming-associated fracture is most likely to occur in this region.

## Introduction

Large bone defects represent an unsolved and growing problem in orthopedics and trauma surgery. Still, the gold standard for filling these defects is autologous bone grafting. Therefore, cancellous bone is often harvested from the iliac crest. This procedure has multiple disadvantages and provides only limited amounts of bone graft, especially in older patients. The Reamer-Irrigator-Aspirator (RIA) technique was developed to overcome the disadvantages of this procedure and obtain larger volumes of bone graft, even in older patients. Previous studies reported harvesting 40–90 ml bone graft by using the RIA technique at the femoral medullary canal^1–4^.

Due to its numerous advantages, the RIA system has become popular^5^. Despite RIA having low morbidity at the harvesting site and none of the complications related to iliac bone crest harvesting^6^, fracturing is still prevalent^7,8^. Surgeons have to decide on the appropriate reaming diameter and whether (or not) an additional or preventive stabilization is necessary based on non-comprehensive recommendations and incomplete literature data. A compromise between safety and collected amount of bone graft needs to be made to ensure no increased fracture risk resulting from the weakened bone.

So far, several experimental and biomechanical studies have reported on RIA and its impact on stability^9–12^. However, to our knowledge, the exact three-dimensional (3D) geometry of reaming has not been investigated. Therefore, this study aimed to systematically investigate the 3D geometry of the reamed bone as a function of the reaming diameter and its influence on the potential fracture pattern.

## Materials and methods

Forty-five fresh frozen (− 20 °C) human cadaveric femora with a mean donor age of 57.1 (range 18–81) years were used. All donors gave their informed consent inherent within the donation of the anatomical gift statement during their lifetime. All experiments were carried out under the relevant guidelines and regulations. Additionally, internal review boards at Science Care (Phoenix, AZ, USA) and the AO Research Institute (Davos, Switzerland) approved the project.

### Study work flow

All specimens underwent computed tomography (CT) scanning (SOMATOM Emotion 6, Siemens Healthcare GmbH, Erlangen, Germany) at 0.6 mm slice thickness. The scans were converted from Hounsfield units to volumetric bone mineral density (vBMD) values using a calibration phantom^13^. Areal bone mineral density (aBMD) was evaluated by generating an artificial X-ray from the CT image via anteroposterior projection, selecting the proximal femoral region including the lesser trochanter, and dividing the bone mineral content with the projected area.

The femora were randomized based on aBMD, age and gender into three equally sized study groups (n = 15) for reaming at a diameter of either 1.5 mm (Group 1), 2.5 mm (Group 2) and 4.0 mm (Group 3) larger than their isthmus. Right and left femora were equally distributed within each group.

Reaming was performed using the novel Reamer Irrigator Aspirator 2 (RIA 2, DePuy Synthes, West Chester, PA, USA). This system can simultaneously ream and irrigate to collect large amounts of cancellous bone for autologous grafting, in contrast to iliac crest harvesting.

Anteroposterior and mediolateral radiographs were taken of each femur in intact state to calculate the isthmus and reaming diameter, followed by standard antegrade trochanteric reaming according to the surgical technique guide. The entry point and distal guidewire position were selected and verified equally for all femora.

Following reaming, all femora were rescanned, with the same parameters as for intact scanning, so that the post-reaming geometry could be generated in a computer. The specimens were then prepared for biomechanical testing based on previous work^12^, and embedded proximally and distally using polymethylmethacrylate (PMMA, Beracryl, W. Troller AG, Switzerland). The femora were then loaded along their anatomical axis in a servo-hydraulic testing machine (MTS Bionix II 858; MTS Systems Corp., Eden Prairie, MN, USA) under 750 N compression load and internally rotated at a rate of 0.25°/s until failure while maintaining the constant axial compression loading. Following biomechanical testing, all specimens were CT scanned so that fracture pattern geometry could be analyzed.

### Data processing and analysis

The generated Digital Imaging and Communications in Medicine (DICOM) data of the three CT scans of each femur in its intact, reamed, and fractured state was imported in Amira software package (version 6.0.0, FEI Company, Hillsboro, OR, USA) for further analysis.

**Figure 1:**
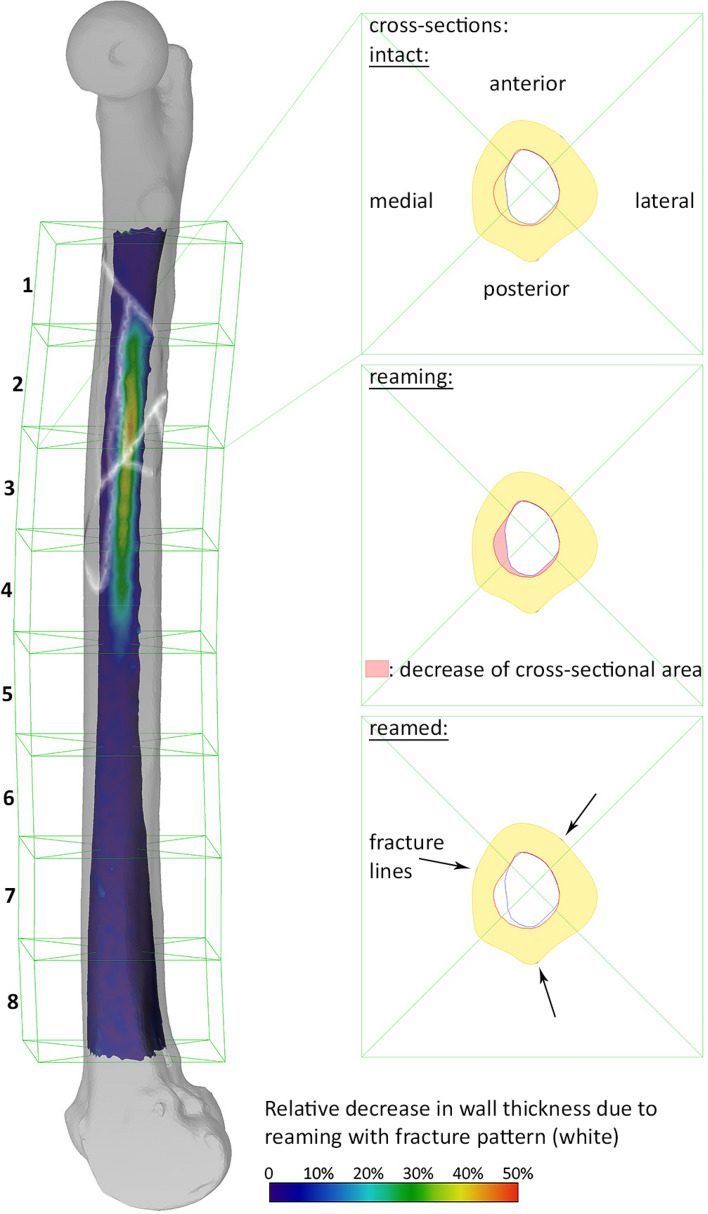
Left: Visualization of the relative decrease in femoral shaft wall thickness post reaming (color-coded) and the fracture pattern (white). The femoral shaft is divided into eighths, and each of those is subdivided into anterior, medial, posterior and lateral quarters (green lines), resulting in 32 regions. Right: Visualization of an exemplary cross-section and the decrease in its cross-sectional area due to reaming.

Following semi-automatic segmentation and virtual fracture reduction of the data from the third CT scan, the image data of all three scans of each femur was aligned to each other via rigid registration. The intact and reamed scans were used to calculate and visualize the relative decrease in wall thickness (%DecWT) post-reaming, considering the complete femoral shaft. The CT scans of the fractured femora were used to extract the fracture pattern and visualize the fracture lines (Fig. 1 left).

The centre line of each femoral shaft was calculated, and the shaft was divided along it into eighths. Each eighth was subdivided into anterior, medial, posterior and lateral quarters, resulting in 32 regions (Fig. 1 left). The region with the maximal relative decrease in wall thickness (Max%DecWT) was determined.

In addition, every eighth was sectioned in 50 axial slices. For every slice, the relative decrease in cross-sectional area (%DecCSA) post-reaming was calculated (Fig. 1 right), and the eighth with the maximal relative decrease in cross-sectional area (Max%DecCSA) was identified (for each separate specimen). The average relative decrease in cross-sectional area per eighth (Mean%DecCSA) was calculated. The eight with the maximal average relative decrease in cross-sectional area (MaxMean%DecCSA) was determined. The absolute and relative decrease in bone volume (DecVol and %DecVol, respectively) was measured for each of the 32 regions. The decrease in bone volume over all 32 regions of the femur resulted in the total volume of harvested bone graft from its shaft during reaming.

An overlap of the fracture pattern with the regions having Max%DecWT, Max%DecCSA and MaxMean%DecCSA was evaluated.

Statistical analysis was performed using SPSS (IBM SPSS, Version 23, Armonk, NY, USA) and GraphPad Prism (GraphPad Prism 7, La Jolla, CA, USA) software packages. The normality of data distribution within each group was checked with the D'Agostino & Pearson Normality Test. One-way Analysis of Variance (ANOVA) and Kruskal–Wallis Test were applied using the corresponding Tukey's Post Hoc Test and Dunn's Post Hoc Test for multiple comparisons to detect significant differences between groups. The level of significance was set to α = 0.05 for all statistical tests.

## Results

The average age of the donors was 55.6 (range 31–73) years in Group 1, 55.5 (range 22–71) years in Group 2, and 60.3 (18–81) years in Group 3. The female/male gender distribution was 3/12 in Group 1, 5/10 in Group 2, and 4/11 in Group 3. No significant differences were detected among the groups for aBMD, endosteal diameter and curvature of the femur, indicating a homogeneous distribution of these architectural parameters, p ≥ 0.49 (Table 1).

**Table 1:** Bone architecture parameters in the three study groups, presented in terms of mean value and standard deviation, and p-values from the corresponding comparisons among the groups.

	aBMD [mgHA/cm^2^]	Endosteal diameter [mm]	Femur curvature [°]
Group 1	0.88 ± 0.14	12.2 ± 2.2	6.6 ± 2.1
Group 2	0.85 ± 0.21	11.5 ± 1.7	6.1 ± 1.9
Group 3	0.84 ± 0.21	11.5 ± 2.4	7.1 ± 1.9
p-value	0.56	0.70	0.49

**Figure 2:**
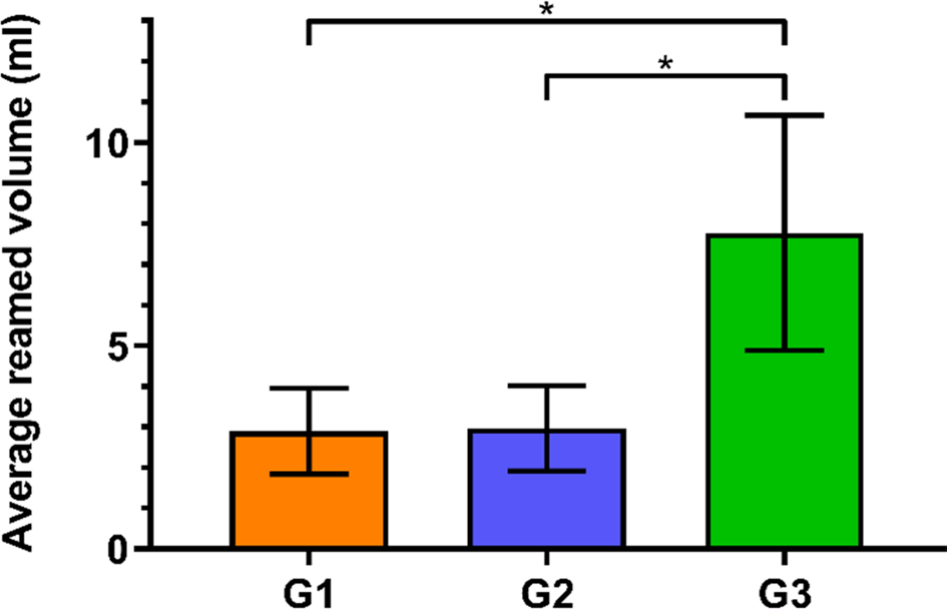
Total calculated volume of harvested bone graft during reaming in the three study groups G1, G2 and G3 in terms of mean value and standard deviation, with stars indicating significant differences.

The total calculated volume of harvested bone graft in the 32 regions during reaming was significantly higher in Group 3 (7.8 ± 2.9 ml, mean ± standard deviation) compared with both Group 1 (2.9 ± 1.1 ml) and Group 2 (3.0 ± 1.1 ml), p < 0.01 (Fig. 2).

Since bone abrasion presented considerable longitudinal variability along the femoral shaft axis (between the separate eighths) and transverse variability within each eighth (eccentricity), a detailed description of the individual parameters is presented hereafter.

A detailed overview of the regions with maximal relative decrease in wall thickness (Max%DecWT) is presented in Table 2. Max%DecWT for Group 1 was with a relative frequency of 71.4% in the second and third eighth, with a frequency of 71.4% in the medial quarter. No clear Max%DecWT could be determined for one femur in this group due to the small amount of harvested bone graft. Max%DecWT was localized in Group 2 with a relative frequency of 73.3% in the third eighth, with a frequency of 73.3% in its medial quarter. For Group 3, Max%DecWT was in 100% of the cases in the third and fourth eighths, with a frequency of 73.3% in the medial quarter. Generally, Max%DecWT was located medially (72.7%) in the third (61.4%), fourth (18.2%) and second (9.1%) eighths of the femur.

**Table 2:** Detailed overview of the absolute and corresponding relative frequencies of occurrence of the maximal relative decrease in wall thickness (Max%DecWT) concerning the regions (as defined by the eighths and quarters) among the three study groups G1, G2 and G3.

Eighth	Anterior			Medial			Posterior			Lateral			Σ per eighth		
	G1	G2	G3	G1	G2	G3	G1	G2	G3	G1	G2	G3	G1	G2	G3
**1**		1 33.3%								1 100.0%			1 7.1%	1 6.7%	
**2**				2 20.0%			2 100.0%						4 28.6%		
**3**				6 60.0%	11 100.0%	9 81.8%						1 100.0%	6 42.9%	11 73.3%	10 66.7%
**4**			2 100.0%	2 20.0%		2 18.2%			1 100.0%		1 100.0%		2 14.3%	1 6.7%	5 33.3%
**5**		1 33.3%												1 6.7%	
**6**		1 33.3%												1 6.7%	
**7**	1 100.0%												1 7.1%		
**8**															
***Σ***	1 7.1%	3 20.0%	2 13.3%	10 71.4%	11 73.3%	11 73.3%	2 14.3%		1 6.7%	1 7.1%	1 6.7%	1 6.7%	14 100.0%	15 100.0%	15 100.0%

Numbers in the three right columns and the bottom row are sums within each separate eighth (and group) and a quarter (and group), respectively.

No fracture could be created for one specimen in each group, with the torque limit set to 200 Nm during biomechanical testing. The extent of the fracture lines over the eighths is visualized and quantified in Table 3. It was observed that Groups 2 and 3 with larger reamed regions were more prone to proximal fractures compared to Group 1.

**Table 3:**
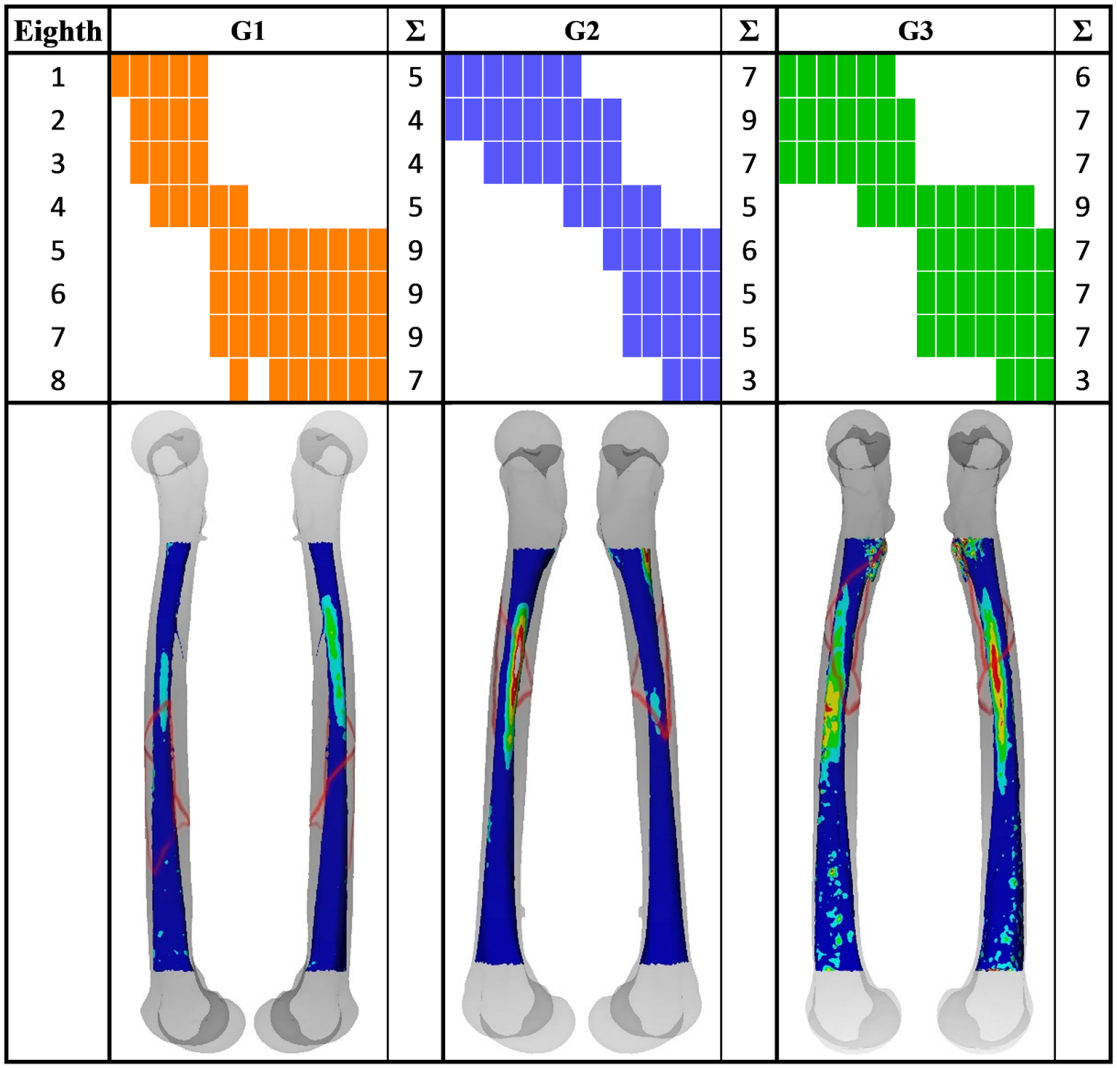
Visualization and quantification of the fracture lines extent over the eighths in each of the three study groups G1, G2 and G3.

Each column represents one femur. In the bottom row, representative pictures of one specimen per group, with relative decrease in femoral shaft wall thickness post reaming (color-coded) and fracture (red lines) are visualized.

An overlap of the eighth indicated with Max%DecWT, and the fracture line was observed with increasing frequency for larger reaming: 38.5% in Group 1, 57.1% in Group 2, and 64.3% in Group 3.

**Figure 3:**
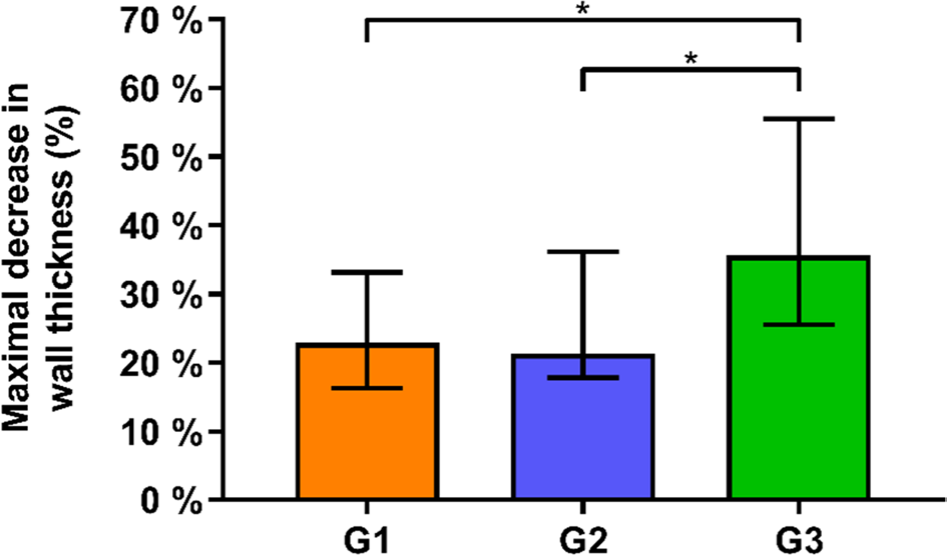
Maximal relative decrease in wall thickness (Max%DecWT) in the three study groups G1, G2 and G3 in terms of median and quartile-one|quartile-three (Q1|Q3) values, with stars indicating significant differences.

The median and quartile-one|quartile-three (Q1|Q3) values for Max%DecWT were 22.9% (16.25|33.13) in Group 1 and 21.3% (17.8|36.1) in Group 2, both being significantly smaller compared to Group 3 with 35.6% (25.6|55.5), p ≤ 0.03 (Fig. 3).

**Figure 4:**
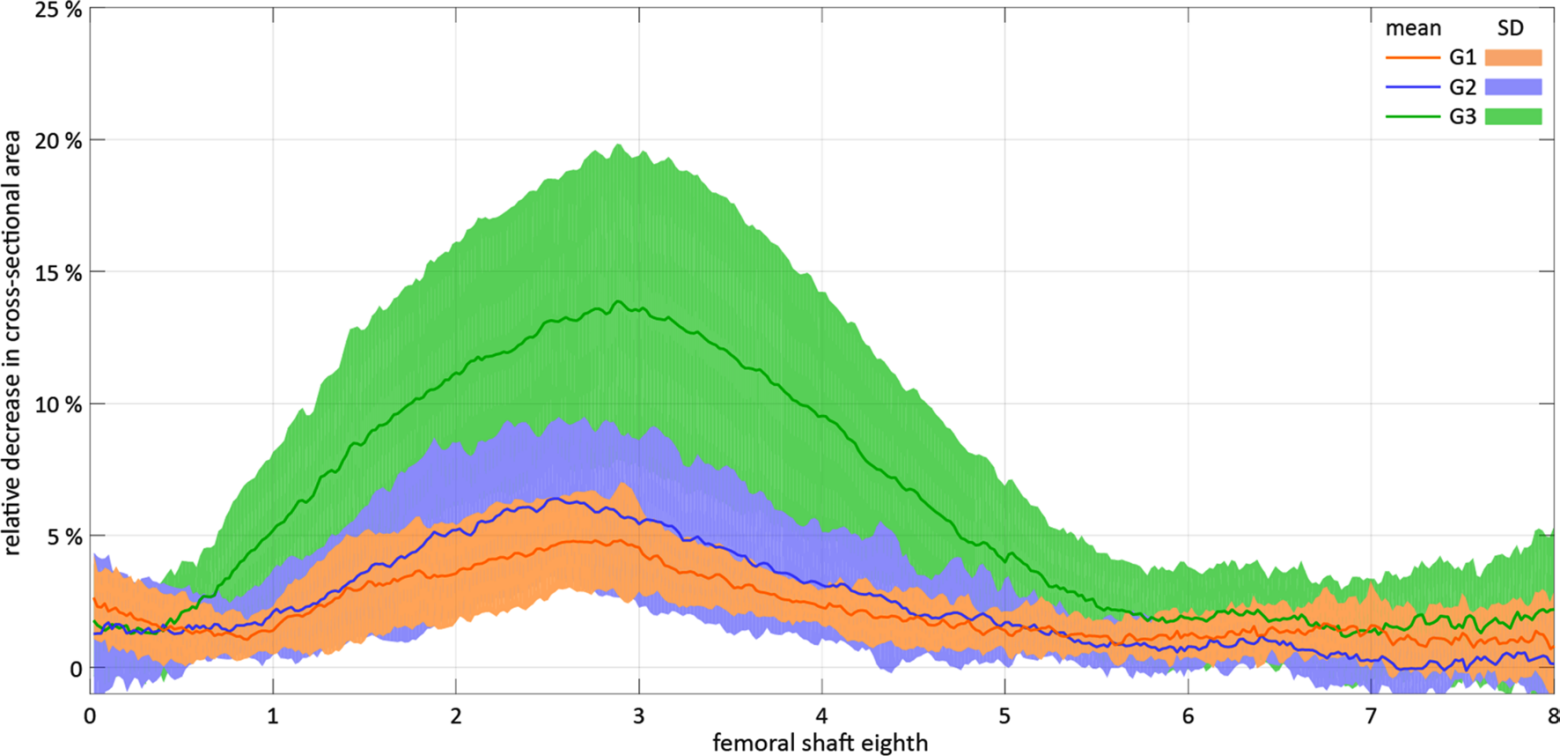
Relative decrease in the cross-sectional area (%DecCSA) in the three study groups G1, G2 and G3 in terms of mean value and standard deviation (SD).

The relative decrease in cross-sectional area (%DecCSA) along the slices of all femoral shaft eighths in the three study groups, visualized in Fig. 4, demonstrates that the main portion of this decrease is located in the region between the 2^nd^ and 5^th^ eighths.

A detailed overview of the eighths with Max%DecCSA and MaxMean%DecCSA is presented in Table 4. The region with Max%DecCSA was located within the third eighth for 60% of the femora in Group 1 and 73% of the femora in Group 2 and Group 3.

**Table 4:** Detailed overview of the distribution of the regions with Max%DecCSA and MaxMean%DecCSA among the eights of the femora from the three study groups G1, G2 and G3.

Eighth	Max%DecCSA			MaxMean%DecCSA		
	G1	G2	G3	G1	G2	G3
**2**	2	2	2	2		
	13.3%	13.3%	13.3%	13.3%		
**3**	9	11	11	10	14	9
	60.0%	73.3%	73.3%	66.7%	93.3%	60.0%
**4**	1	1	2	2		6
	6.7%	6.7%	13.3%	13.3%		40.0%
**5**		1			1	
		6.7%			6.7%	
**7**	2					
	13.3%					
**8**	1			1		
	6.7%			6.7%		
***Σ***	15	15	15	15	15	15
	100.0%	100.0%	100.0%	100.0%	100.0%	100.0%

An overlap of the eighth indicated with Max%DecCSA, and the fracture line had a higher frequency of 50.0% for Group 2 and Group 3 than Group 1 (42.9%). An overlap of the fracture line and the eighth indicated with MaxMean%DecCSA was observed with increasing frequency for larger reaming: 35.7% in Group 1, 57.1% in Group 2, and 64.3% in Group 3.

**Figure 5:**
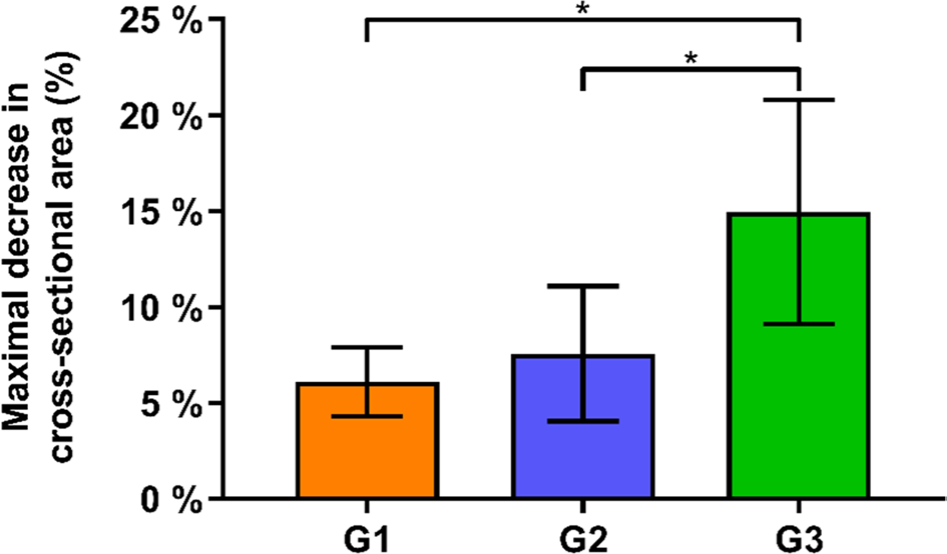
Maximal relative decrease in cross-sectional area (Max%DecCSA) during reaming in the three study groups G1, G2 and G3 in terms of mean value and standard deviation, with stars indicating significant differences.

The maximal relative decrease in cross-sectional area (Max%DecCSA) was significantly higher in Group 3 (15.0 ± 5.8%) compared with both Group 1 (7.6 ± 3.5%) and Group 2 (6.1 ± 1.8%), p < 0.01 (Fig. 5).

**Figure 6:**
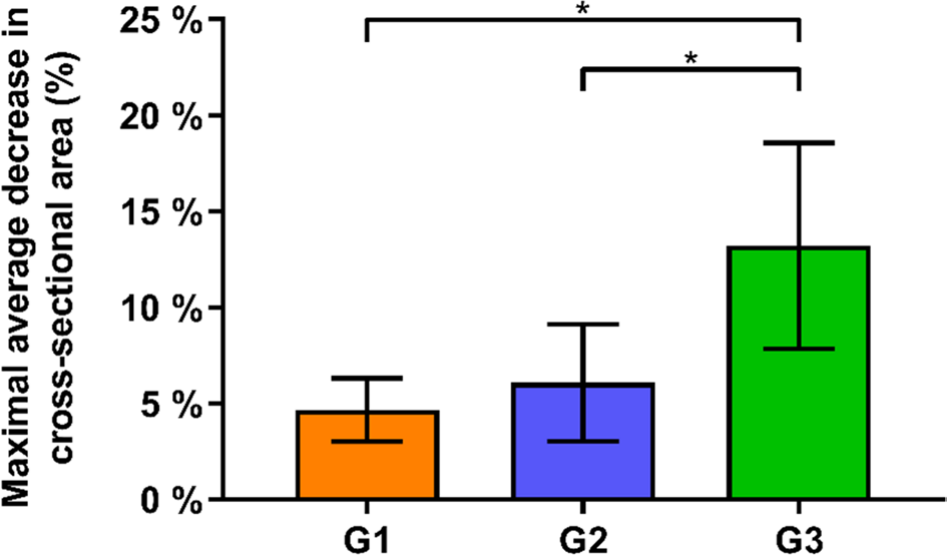
Maximal average relative decrease in the cross-sectional area (MaxMean%DecCSA) during reaming in the three study groups G1, G2 and G3 in terms of mean value and standard deviation, with stars indicating significant differences.

Similarly, the maximal average relative decrease in cross-sectional area MaxMean%DecCSA was significantly higher in Group 3 (13.2 ± 5.4%) compared with both Group 1 (4.7 ± 1.7%) and Group 2 (6.1 ± 3.0%), p < 0.01 (Fig. 6).

**Figure 7:**
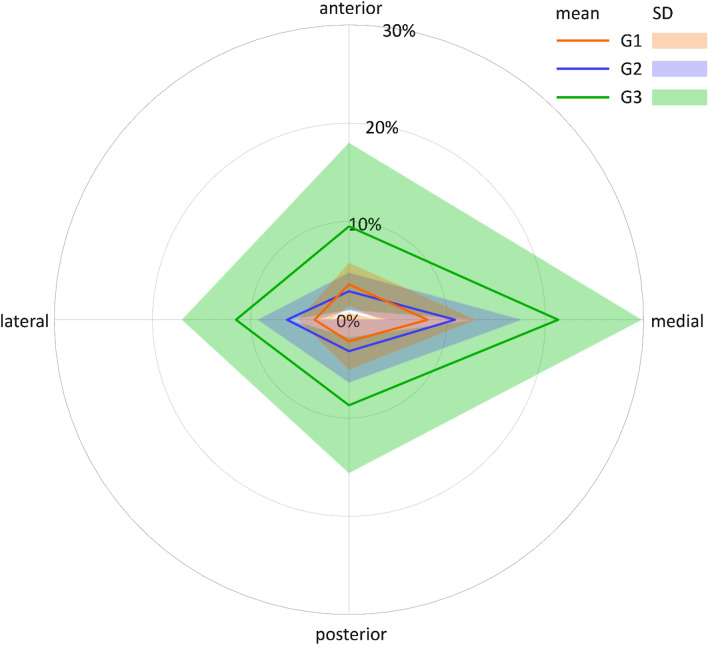
Net diagram visualizing the eccentricity of reaming in the third eight of the femora from the three study groups G1, G2 and G3 in terms of mean value and standard deviation (SD) of the relative volume decrease (%DecVol) in each of the four quarters (anterior, medial, posterior and lateral).

**Figure 8:**
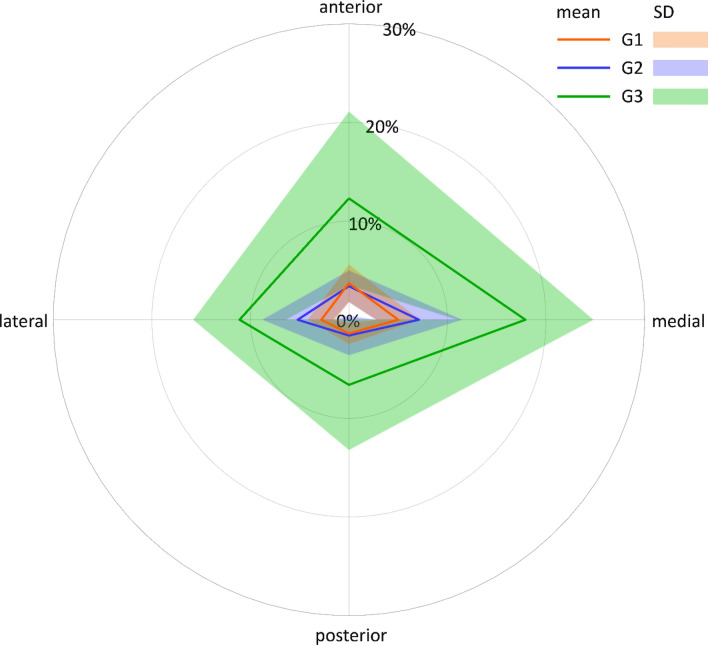
Net diagram visualizing the eccentricity of reaming in the fourth eight of the femora from the three study groups G1, G2 and G3 in terms of mean value and standard deviation (SD) of the relative volume decrease (%DecVol) in each of the four quarters (anterior, medial, posterior and lateral).

The relative volume decrease (%DecVol) at the third and fourth femoral shaft eighths—representing the regions with most pronounced reaming—is visualized in Figs. 7 and 8, respectively, where the eccentricity of reaming becomes visible in all three study groups exhibiting a comparable eccentricity pattern. However, whereas in the third eighth the eccentricity is pronounced and focused on the medial quarter (Fig. 7), in the fourth eighth its focus is distributed to a certain extent between the medial and the anterior quarters (Fig. 8).

Biomechanical testing demonstrated a significant lower torque at failure in all reamed groups compared to the corresponding control groups from a previous study (Table 5). Interestingly, the torsional stiffness was only reduced significantly in Group 3 (compared to its control group). The entire results of the biomechanical analysis can be found in the work of Schmitz et al.^12^.

**Table 5:** Results of the biomechanical testing presented in terms of mean values and standard deviation according to Schmitz et al.^12^.

	Paired groups					
	G1		G2		G3	
	Reamed	Control	Reamed	Control	Reamed	Control
Torsional stiffness [Nm/°]	9.7 (1.9)	10.0 (2.1)	9.5 (1.8)	10.0 (2.2)	8.5 (1.6)	9.9 (2.0)
Failure torque [Nm]	125.8 (24.5)	140.5 (33.1)	105.4 (21.2)	133.3 (29.1)	102.9 (19.5)	136.4 (26.4)

## Discussion

There are controversial discussions in the literature about the occurrence rate and cause of femoral fractures following bone harvesting with the RIA system. Lowe et al. reported that after harvesting with the RIA system, a thinning of the anterior cortex resulted in femoral fractures^7^. Qvick et al. and Calori et al. concluded that RIA is a safe procedure as very few fractures were observed in their reported study cohort^5,14^. The reaming diameter is a crucial factor related to the cortex weakening and the occurrence of those fractures. However, the authors have not reported which reaming diameter was used and how it was related to the femoral isthmus^5,14^.

Besides the reaming diameter and the resulting residual cortex thickness, it is also essential to know whether the inner cortex is removed uniformly (centric reaming) or asymmetrically (eccentric reaming) during reaming. Lowe et al. support this consensus as they found that eccentricity is an essential factor for weakening the bone^10^.

Several studies have investigated the biomechanics of reaming^10–12,15^. To our knowledge, the current work is the first one to systematically investigate the 3D geometry of femoral reaming in a more extensive series of human cadaveric specimens. CT scanning was performed before and after reaming to measure the eccentricity and thickness of the removed cortex reliably. Furthermore, in contrast to previously published work^10^, the scans were not evaluated in just singular slices but over the complete region of reaming.

The results from our study suggest that the guidewire and the stiffness of the reamer are crucial for setting the reaming location. Pre-bending the wire so that it can be centered in the medullary canal has only a minor effect. The reaming trajectory arcs start from the entry point made at the greater trochanter to the intercondylar notch. Its apical point is located medially to anteromedially within the third to fourth eighth of the femoral shaft.

Even attempting to ream centrally, the apical point was still the most substantial region of bone removal with anteromedial reaming.

The other crucial factor is the inner shape of the medullary cavity, which may promote eccentric reaming due to its irregular geometry and the resulting uneven contact surface of the reamer head to the bone. The inner cross-section is approximately circular but with a tangential flattening on one side. In that case, the reamer will permanently remove this flattening because it represents the smallest cross-sectional area for removal with the minor mechanical resistance and applied energy, regardless of where the guidewire is located. It should also be considered that reaming cross-sectional area is not directly proportional to reamer diameter during equidistant step increase to larger reamer head diameters. For example, if the head increases from 12 to 13 mm, the area increases by 19.6 mm^2^ (17.4%), but if increases from 17 to 18 mm, then the area increases only 27.5 mm^2^ (12.1%).

Regarding the criterion for selection of maximal reamer head size (in millimetres) larger than the isthmus, a relevant question would be whether it should not be more reasonable to determine the relative thickness decrease of the cortex as measure for this selection. In the case of a thin cortex, removing, e.g. 1 mm could produce a much more significant weakening than in a thicker cortex.

We also found significantly higher amounts of harvested bone in the group with the largest reaming diameter in this investigation. Those volumes cannot be compared with the amounts reported in clinical settings^1–4^ because we calculated the decrease in volume only of the femoral shaft over all 32 regions. In our previous biomechanical study, we were able to show significantly higher mean amounts of bone graft in Group 3 (26 g) compared to Group 1 and Group 2 (9 g and 14 g, respectively)^12^. Nevertheless, even these volumes are minimal compared to clinically achievable quantities.

One reason is that in the present study, only the medullary canal of the femora was reamed; in the clinical setting, the femoral condyles are also addressed, which significantly increases the bone graft volume. Furthermore, coagulated blood/bone marrow helps increase the quantity in clinical application, which is not the case in an in vitro scenario.

This study has limitations similar to those inherent to all cadaveric investigations, incapable of completely simulating the in vivo situation. A limited number of specimens at a sample size of fifteen per study group were used, restricting the translation to generalized clinical applications.

## Conclusion

In summary, it was found that regardless of the reaming diameter of the novel Reamer-Irrigator-Aspirator 2 system, the most substantial relative decrease in femoral shaft wall thickness occurred medially between the second and fourth eighths of the femoral shaft. As the diameter of reaming increases, an overlap of the fracture line with the region featuring maximal relative decrease in wall thickness and the maximal average relative decrease in the cross-sectional area becomes more frequent. This suggests that a reaming-associated fracture is most likely to occur in this region. Future device optimizations may attempt to incorporate this information to allow for more targeted local reaming.
